# Supplementary material for: Identification of photoperiod-induced specific miRNAs in the adrenal glands of Sunite sheep (Ovis aries)
Source: Front Vet Sci. 2022 Jul 22;9:888207. doi: 10.3389/fvets.2022.888207 (PMC9354845; doi:10.3389/fvets.2022.888207)
Supplement: Supplementary file 1 [file Data_Sheet_1.ZIP › Supplementary file/miRDeep2 file.zip!/miRDeep2 file/Know_Structure/SP42A2/oar-mir-323c.pdf]

miRBase precursor : oar-mir-323c
Total read count : 236
oar-miR-323c read count : 235
remaining reads : 1

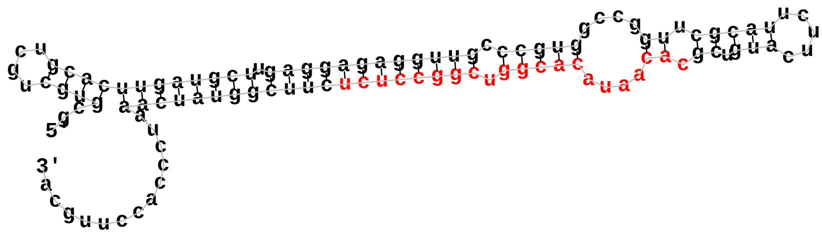

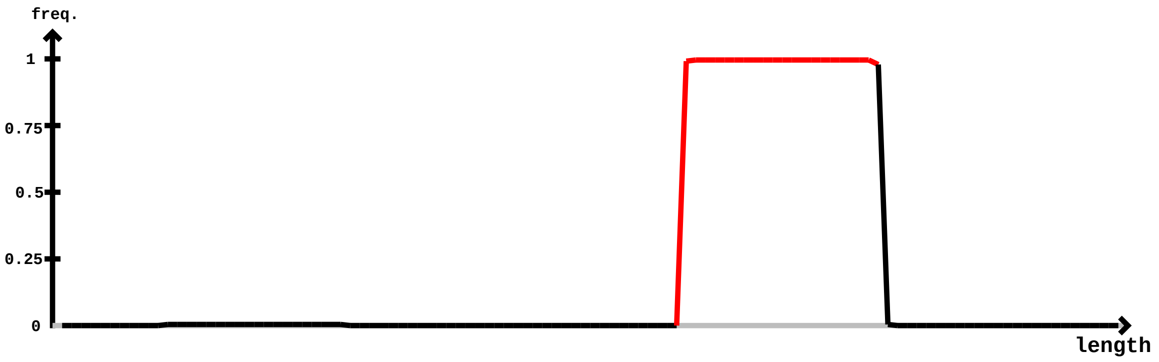

oar-miR-323c

```
5'- gcgugcugcugcacuugaugcuugaggagagguugcccguggccgguucgcauucuucaugucgcacaauacacggucggccucucuucgguaucaaaucccaccuugca   -3'   exp
    ..((((....))))((((((((.(((((((((((((.(((((....((.(((((.....))).)).)).))....))))).)))))))))))))))))))).............   reads   mm   sample
    ..........gcacuugaugcuugaggag.................................................................................   1       0    seq
    ................................................................cacaauacacggucggccuc..........................   4       0    seq
    ................................................................cacaauacacggucggccucu.........................   229     0    seq
    ................................................................cacaauacacggucggccucuc........................   1       0    seq
    .................................................................acaauacacggucggccucu.........................   1       0    seq
```
